# Does Servant Leadership Stimulate Work Engagement? The Moderating Role of Trust in the Leader

**DOI:** 10.3389/fpsyg.2022.925732

**Published:** 2022-07-05

**Authors:** Guangya Zhou, Rani Gul, Muhammad Tufail

**Affiliations:** ^1^College of Education, Anyang Normal University, Anyang, China; ^2^Departmental Ethical Committee, Faculty of Education, University of Malakand, Chakdara, Pakistan; ^3^Institute of Business Studies and Leadership, Abdul Wali Khan University, Mardan, Pakistan

**Keywords:** work engagement, leadership styles, social exchange theory, servant leadership, moderator

## Abstract

A positive leadership style can promote work engagement. Using social exchange theory, this study examines the impact of employee leadership styles on work engagement. In addition, the link also considered the mitigating role of trust in leaders. Preliminary data were collected from the educational and non-educational staff of the Business Management Sciences and Education Department at different universities. We collected responses from 242 employees from selected universities using the purposive sampling technique. We tested the proposed hypothesis using linear regression. Research has shown that there is a positive link between employee leadership and work engagement. When trust in leaders as facilitators was introduced, the relationship between leadership and work engagement was relaxed to increase trust in leaders. Practical and theoretical contributions to the study were provided with recommendations for further study.

## Introduction

For long-term development, organizations need to identify factors that encourage employees to actively participate in their work (Den Hartog and Belschak, 2012). Employees who follow normal work routines are productive, enjoyable, efficient, and proactive (Tims et al., 2011). Trust in the organization is recognized as one of the key factors in increasing employee involvement (Buckley, 2011). Management needs to be confident in their employees, as they are expected to engage in day-to-day operations. Excessive control oversight and enforcement can discourage employees from participating in their duties. Integrity affects trust between leaders and followers Ahmad et al. (2022). The impact of integrity on the concept of trust was given to followers honestly: Trusting the leader. Attention has a higher level of integrity (Schoorman et al., 2007). Non-profits (such as universities) face many obstacles, i.e., decreased sales and limited funding (McDonald, 2007) and lack of commitment. Such problems reinforce the need to overcome these obstacles and require a more flexible and skilled workforce for sustainability. Leadership plays an important role in observing the behavior of extra roles. Leadership styles, whether profit or non-profit, have common characteristics (Akingbola, 2013; Gul et al., 2022), relying on outstanding leadership, a style in which these challenges can be discussed (Aboramadan, 2018). Given the academia as a non-profit organization, employee leadership is an appropriate approach (Ortiz-Gomez et al., 2020). Employee leaders are followers who prioritize profits, focus on selfless value (Banks et al., 2018), and consider themselves as other workers (De Clercq et al., 2014).

The concept of servant leadership was created by Greenleaf (1977), whose purpose was to serve rather than gain power (Luthans and Avolio, 2003; Aboramadan et al., 2022). Employee leaders are said to act as agents (Van Dierendonck, 2011) and adopt employee-centric tactics. Such attributes benefit workers, businesses, and communities (Reinke, 2004). Many organizations also wanted servant leadership as the dominant leadership style (e.g., Van Meter et al., 2016; Lumpkin and Achen, 2018). Hoch et al. (2016) encouraged servant leadership to explain more diversity than other styles (e.g., transformative, ethical, or authentic leadership styles), but, because of its early stages, called for more empirical research (Donia et al., 2016). Social Exchange Theory (SET) explains how serving leaders affect their followers. It suggests that interdependent individuals respond to positive or negative behavior (Blau, 1964). Reciprocity is based on trust (Lioukas and Reuer, 2015) and leads to loyalty and commitment (Cropanzano, 2005) and sustainability of relationships (Konovsky and Pugh, 1994). For example, employee leaders can increase trust in their employees by demonstrating fairness, morality, and honesty (Ling et al., 2017). Confidence increases because of the credibility and discernment of the leader (Greenleaf, 1977). Positive leadership behavior (e.g., servant leadership) naturally instills trust in followers (Goh and Low, 2014), and employees invest more energy to achieve goals and results (Saks, 2006) and encourage greater involvement (Ahmad et al., 2019).

Trust in leaders has been extensively studied and associated with a variety of work outcomes (DeConinck, 2011). Trust has been categorized as an important aspect of various leadership theories and has been found to empower subordinates and verify that leaders are confident in their skills and capabilities (Solomon and Flores, 2003). In this regard, Ötken and Cenkci (2012) warned of a lack of research and suggested conducting research on trust in executives as a moderating variable. Taking these recommendations and discussions into account, this study focuses on senior management because research is about leadership style and beyond. Managers are motivated by intrinsic rewards (Kuvaas, 2006), peer relationships, top management (May et al., 2002), and the obvious opportunities (Thompson and Heron, 2005). Highly skilled and usually engaged in multifaceted tasks (Agarwal, 2014), top managers execute organizational strategies, drive change, create operational environments, and motivate their subordinates (Agarwal, 2014). Delmestri and Walgenbach, 2005). Organizations rely primarily on managerial ingenuity and revolution (Dutton et al., 1997). Therefore, trust in the leader can ease the relationship. Confidence in leaders as moderating variables lacks research (Ötken and Cenkci, 2012; Gul et al., 2021a,b,c) and requires further empirical research. Haq et al. (2021) also called for further research on individual outcomes and the moderating role of management confidence in work engagement. In addition, Borst et al. (2020) suggested that research was conducted primarily in developed countries, with significantly less in developing countries, and further research was recommended in developing countries (Middle East, Africa, and South Asia). To ensure that there are homologous results in the proposed region, one can see if the intended effect is replicated across the country. In this regard, it was confirmed that only 24% of South Asian employees are involved in their work. Previously, Resick et al. (2011) suggested that different leadership styles in different countries can have different effects. While maintaining these recommendations, this survey focuses on one country in South Asia, Pakistan.

## Literature Review

### Servant Leadership and Employee Engagement

Leadership's function as an antecedent to employee engagement has been recognized in the previous study (Shuck and Herd, 2011). However, other leadership styles, for example, authentic, spiritual, and transformational leadership styles, have been extensively studied (Walumbwa et al., 2010; Ahmad and Gul, 2021). Although servant leadership has some similarities with these leadership styles (Penger and Cerne, 2014; Schaufeli, 2015), it is distinct because it is a more comprehensive approach that encompasses all aspects of leadership. Likewise, empirical research reveals that servant leaders are those that commit themselves to giving chances for their followers to build new skills and knowledge, as well as supporting them to achieve their objectives *via* the use of their intellectual talents and capacities (Walumbwa et al., 2010; Gul et al., 2021a,b,c). Employees keep themselves engaged in productive activities when receiving such positive encouragement and support (Hakanen et al., 2017). Work engagement can be defined as “*a good, gratifying state of mind associated with labor that is marked by energy, devotion, and absorption*”. Vigor implies higher energy and flexibility, willingness to exert more effort, and determination. Dedication means a sense of commitment, eagerness, and challenge. While absorption refers to full concentration and absorption in work. Work engagement entails these three facets, yet confirmed a single factor, and this study also counted work engagement as a uni-dimensional construct. Positively engaged employees in their work result in lesser wastage of existing resources. Servant leadership diagnose followers' qualities (van Dierendonck and Nuijten, 2011), and followers are motivated (Schaufeli and Bakker, 2004). Followers show more dedication when their personal needs are addressed (Page and Wong, 2000; Yan et al., 2020). In academic settings, the influence of servant leadership has been identified (Aboramadan et al., 2022).

Therefore, employees who work in this style of management are expected to increase their commitment to their daily work. However, there are few studies on this association, and recent studies suggest more evidence (e.g., Alafeshat and Aboud, 2019). Therefore, we proposed the following hypothesis:

*H1:There is a significant relationship between servant leadership and employee work engagement*.

### Trust in Leader and Work Engagement

Trust can be defined as “*A psychological condition characterized by the goal of tolerating vulnerability based on favorable expectations of another's intentions or behavior*.” (Rousseau et al., 1998, p. 395; Ali and Zafar, 2021). Trust in the leader has been an important area for research studies and has been studied concerning job antecedents and work outcomes (DeConinck, 2011), for example, Organizational citizenship behavior (Choong et al., 2019; Ayub et al., 2021a,b), organizational commitment (Abbas et al., 2021), job satisfaction (Fard and Karimi, 2015), job performance (Zhu et al., 2021), and proactive behavior (Parker et al., 2006).

For work engagement, trust is one of the key indicators. Trust allows employees to be productive and enthusiastic about their work (Agarwal, 2014). When trust is low, employees spend more time protecting themselves. Trust has been discovered as a reason why certain employees can perform their duties properly and act discretionarily without compensation. This is similar to the concept of “employees traveling extra distances” that is characteristic of engaged workers (Abbas et al., 2020). According to SET (Blau, 1964), recognition of trust in leaders can develop mutually. In other words, employees respond to the treatment they receive from within the organization or from leaders. He further suggested that social or economic principles form the basis of any commutation relation. Like economic benefits, social exchange predicts future benefits, but the nature is not yet clear as employees are considering how they were valued. Therefore, trust in leaders is important for maintaining social exchange, as it creates a commitment to show positive work attitudes and behaviors (Gul et al., 2021a,b,c). For example, during difficult times or increased workload, employees show discretionary behavior related to commitment and repayment to the organization. They are convinced that the recognition of leaders' insights and skills will bring more benefits to both the organization and its employees (Spreitzer and Mishra, 2002). This awareness allows employees to focus on the tasks they need, rather than other issues (Mayer and Gavin, 2005). Trust in leaders is a driving force that motivates employees to focus on their work and is a condition of a serious working environment. Therefore, we proposed the following hypothesis:

*H2: Trust in a leader has a significant impact on work engagement*.

### The Moderating Role of Trust in Leadership

Trust is a vital drive for leadership efficacy and has been acknowledged for the prosperity of any organization. Trust has been classified as affective or cognitive (Azizi et al., 2021). Affective trust, based on the social exchange process, is related to the emotional tie between leader and subordinates, while the latter originates from the characteristics of a leader (Su et al., 2021). The level of trust between leaders and subordinates determines the strength of a relationship. Trust is the willingness to exchange and takes place when the employees believe that exploitation would not take place and collaborative relationships would be exercised. The collaborative relationship can be developed by leaders based on integrity and authenticity (Avolio et al., 2004). Leaders' consistent fair actions develop a healthy atmosphere (Coxen et al., 2016; Abbas et al., 2019), and such actions help in the development of positive behavior (Dirks and Ferrin, 2002; Gul et al., 2021a,b,c). When employees find their leader/supervisor trustworthy, it positively affects their well being (Su et al., 2021), and employees would be more engaged in their duties (Wang and Hsieh, 2013). Moreover, when individuals find trust and support from their leaders and growth in careers (Saks and Gruman, 2018), such personal progress and development might be expressive and result in emotional engagement and enthusiasm.

Trust has been widely studied as a moderator (see, Chang and Wong, 2010; Bal et al., 2011; NeJhaddadgar et al., 2020) due to the reason for the main pillar of the relationship (McAllister, 1995). Keeping in view the same, it is proposed that servant leadership will influence the employees' behavior in organizations (Gul and Khilji, 2021). As discussed earlier, a leader can develop and sustain a good relationship and urge employees to refund the organization in the same way. This study focuses on the moderating role of trust in a leader in a link between servant leadership and work engagement. Trust is a vital element due to daily interaction and work engagements. Trust confirms the cooperation is organizations (Misztal, 1996; Khan et al., 2020). Servant leadership promotes a favorable working environment that urges employees to show positive behavior. Similarly, trust in the leader also plays the same role. It is, therefore, assumed that servant leadership, when paired with trust in the leader, may strengthen the said relationship, and the employees may be found more engaged in their duties. Thus, we proposed the following hypothesis:

*H3: Trust in a leader would moderate the link between servant leadership and work engagement such that the relationship would be stronger for higher trust in the leader*.

## Methodology

We collected data from faculty members and non-faculty members working at the universities. The target population was recruited from the University of Khyber Pakhtunkhwa, located in a province of Pakistan. According to the Khyber Pakhtunkhwa has 40 public and private university/degree awarding institutions. Data were collected from two faculties of the selected university, namely, the Faculty of Management Sciences and the Faculty of Education. Different organizations deal with different external competitive pressures and have a positive impact on employees (Hodson, 2002; De Clercq and Belausteguigoitia, 2017; Gul et al., 2020). Thus, for this reason, we collected data from the same organizations, which helped us to avoid the perception and evaluation of different affecting factors across organizations.

English is the mode of official correspondence in both public and private organizations in Pakistan (Abbas and Raja, 2019); therefore, it was unnecessary to translate the questionnaire into the national language as the employees who were targeted for data collection were well aware of English. Employees were approached by getting permission from the Registrars of the universities. The context and aims of the study were cleared to them, and then, the questionnaires were distributed. The offices of the respective respondents were visited and were requested to take part in the study. The collected data relied on a survey instrument we collected from 237 employees. Respondents were approached by adopting the purposive sampling technique. The purposive sampling technique allows researchers to follow their judgment and information. The general threshold of response above 50% of the distributed questionnaire is desirable (Babbie and Benaquisto, 2009). Among the distributed questionnaires, we got a 64.2% response rate, an appropriate percentage in the Asian region (Abbas et al., 2014; Tufail et al., 2017). The data were collected in two rounds. In the first round, responses against servant leadership and work engagement were recorded, while in the second round, responses against trust in leader were recorded. Details about the distributed and received questionnaires are provided in Table 1. For the analysis, 22 questionnaires were discarded, as they somehow were not completed with all aspects. A complicated question was added to the adopted questionnaire to confirm the quality of the responses prescribed by Torres et al. (2017). The respondents who answered this question wrongly were not included in the analysis. The demographic analysis (Table 2) resulted that among the employees who took part in the study, the average age was 33.7 years, 6% of the employees hold master's degree, 71% of the employees hold MS degree, 20% of the employees hold PhD, and only 3% of the employees hold post-doctorate. Among the respondents, the average tenure for the current organization was 5.2 years and 72% were men.

**Table 1:** Break down of sample size.

**Particulars**	**No. of the questionnaires distributed**	**Percentage (%)**
Composition of questionnaire
Distributed	300	100
Completed	242	80.67
Discarded	22	7.33
Not received	36	12

**Table 2:** One-way ANOVA.

**Demographic**	***F***	***P***
Age	1.36	0.17
Gender	0.35	0.81
Education	2.97	0.03
Experience	0.73	0.60

### Measures

All the study variables were measured through questionnaires adopted from previous studies. Servant leadership was measured through 28 items developed by Liden et al. (2008). The responses were recorded regarding the servant leadership from employees who work under the direct supervision of the manager/leader rather than the general manager of the organization. Employee work engagement was measured through 9 items scale developed by Schaufeli et al. (2007). Responses regarding trust in leaders were recorded through 6 items scale developed by Podsakoff et al. (1990). All the scales were on a five-point Likert scale. Discouraging the threat of “guessing” the order of the items was counterbalanced (Darvishmotevali and Ali, 2020).

### Control Variable

One-way ANOVA was run to check the significance of demographic variables. We found gender as significant, and thus, it was used as a control variable.

### Statistical Analysis

Multiple regression analysis was run to test the proposed hypothesis 1 (there is a significant relationship between servant leadership and employee work engagement) and hypothesis 2 (trust in a leader has a significant impact on work engagement. Also, moderated regression analysis was run to test the third hypothesis (trust in a leader would moderate the link between servant leadership and work engagement such that the relationship would be stronger for higher trust in the leader). In our model, trust in leadership was proposed as a moderating variable and was hypothesized to strengthen the relationship between servant leadership and work engagement.

### Validity Analysis

Confirmatory factor analysis was carried out before testing the proposed hypothesis. Three-factor and one-factor models were run to validate the distinctiveness of the study variables. Table 3 predicts that the three-factor model was fit than the one-factor model: χ^2^/*df* = 2.57; IFI = 0.91; TLI = 0.92 CFI = 0.91; RMSEA = 0.05. These values confirmed the discriminant validity, and no common method bias was found.

**Table 3:** Model fit.

**Model**	**χ2**	***df***	**RMSEA**	**CFI**	**IFI**	**TLI**
Three Factors (Hypothesized)	1887.23	732	0.04	0.93	0.92	0.93
One factor (All items on a single factor)	2941.75	738	0.08	0.71	0.73	0.71

## Results

Table 4 shows the correlation among the study variables and found that there are significant relationships between variables, confirming the initial support for the proposed hypotheses.

**Table 4:** Correlation and reliabilities.

**Variable**	**1**	**2**	**3**
SL	(0.81)		
TL	0.15**	(0.85)	
WE	0.40**	0.35**	(0.78)

N = 242. “*” means the correlation is significant at the 0.05 level (2-tailed). “**” means the correlation is significant at the 0.01 level (2-tailed).

Correlation is significant at 0.01 levels (2-tailed). Correlation is significant at 0.05 levels (2-tailed). Alpha values are given in parentheses.

### Hypotheses Testing

Table 4 exhibits regression analysis. The first hypothesis proposed that ethical leadership has a positive relationship with work engagement. The results confirmed the said relationship (β = 0.53, *p* < 0.05) and thus supported the hypothesis. Furthermore, the second hypothesis, trust in the leader has a significant relationship with employee work engagement, was also confirmed (β = 0.68, *p* < 0.05).

Finally, the third hypothesis states that trust in leader moderates the relationship between servant leadership and work engagement. The results Table 5 supported supported that trust in the leader moderates the said relationship. The combined effect of servant leadership and trust in the leader was found significant (β = 0.38, *p* < 0.05). Furthermore, Δ*R*^2^ was found 0.07, a minute but significant effect.

**Table 5:** Moderating analysis.

**Moderator trust in leader**	**Dependent: Work engagement**			
	**β**	**SE**	**LLCI**	**ULCI**
Constant	1.81**	0.18	1.82	2.43
Education	0.29**	0.15	0.03	0.52
TL	0.31**	0.09	0.85	0.50
SL	0.58**	0.08	0.41	0.64
TL × SL	0.15**	0.09	0.55	0.22
Δ*R^2^*	0.07**			
*F*	15.54			

Two stars ‘**' denote that the corresponding variable is significant at 5% level.

**Figure 1:**
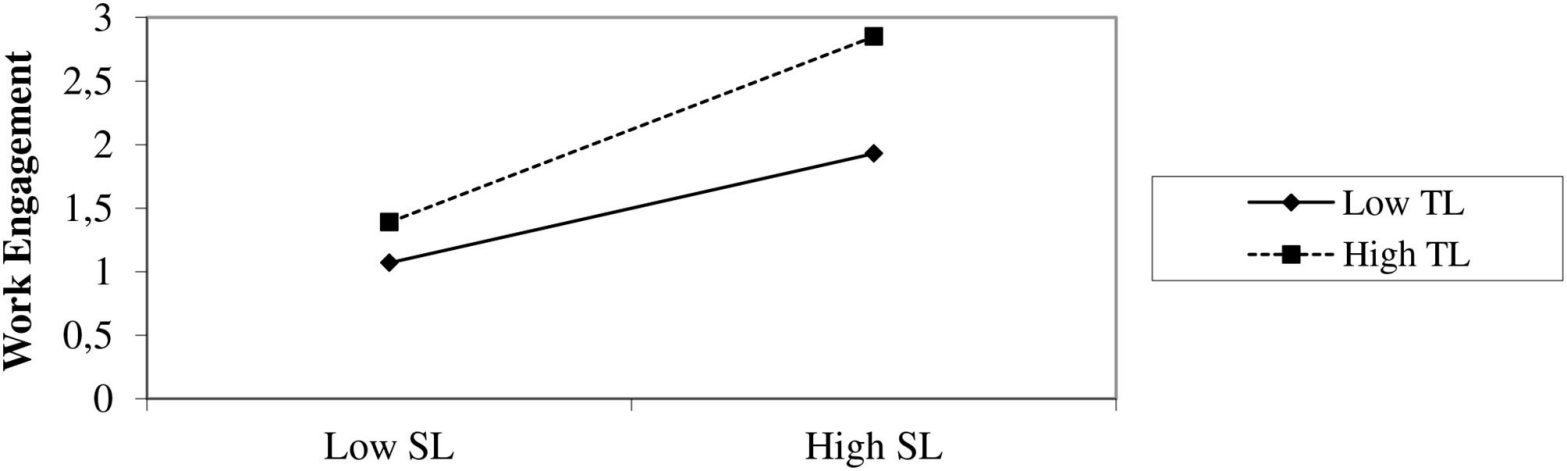
Moderating role of trust in leader between servant leadership and work egagment.

Figure 1 illustrates the moderating effect of trust in a leader. It elaborates that the servant leadership–work engagement relationship was strengthened for higher trust in the leader.

## Discussion

As an important factor for organizational effectiveness, every organization wants to have engaged employees. Employee engagement has been investigated at an individual level, at the organizational level, and even at the organizational analysis level (Bakker et al., 2008). Similarly, different leadership styles have also been investigated concerning work engagement, for example, transformational leadership (Amor et al., 2020), authentic leadership style (Oh et al., 2018), ethical leadership (Ahmad and Gao, 2018), and paternalistic leadership (Öge et al., 2018). These studies have declared the positive effect of leadership styles on work engagement. Servant leadership shares some features with these leadership styles, and its dimensions are inclusive in supporting employees' development, which is favorable for skills development, enhancing abilities, and productivity. Our results concluded that servant leadership has a positive impact on employee work engagement. The relationship between servant leadership and work engagement is based on the social exchange theory. It proposes that reciprocity supports a positive working environment. Trust sustains social exchange relationships (Konovsky and Pugh, 1994). Thus, working in such a supportive environment employees would be more involved in their work.

If employees believe that their leaders are trustworthy and the decision taken by them would be in the best interest of both individuals and the organization, they would be more willing to engage in their duties (Buckley, 2011). Our study confirmed that a relationship between leader and subordinates, if trustworthy, would endorse engagement. Such engagement will be based on the trust in the leader. Such perception is developed by psychological safety (Maximo et al., 2019). For example, if a relationship is characterized by trust, individuals will expect fair treatment, and a psychologically harmless environment is likely to support work engagement. In such an environment, employees are more innovative and involved in their duties. Robinson et al. (2004) suggested that engagement is a two-way process. When employees find their leaders honest, they repay in the form of engagement. A sense of engagement is developed (Cropanzano and Mitchell, 2005). Previous studies also concluded the significant impact of trust and job outcomes (Buckley, 2011).

Regarding the moderating role of trust in a leader, it was concluded that trust in a leader significantly moderates the relationship between servant leadership and work engagement. It is confirmed that trust in a leader has a significant impact on job outcomes (DeConinck, 2011). Trust is a vital aspect of leadership theories (Ötken and Cenkci, 2012). Our study points out that when employees trust their leaders, they would be involved in positive behavior irrespective of the leadership style; rather, employees would show a sense of responsibility and commitment. Trust in a leader, when paired with a servant leadership style, strengthened the link with work engagement.

## Conclusion

This study adds to the area of leadership and work engagement by examining the relationship between servant leadership and work engagement, along with the moderating role of trust in leaders in academic settings. With Social Exchange Theory as the underpinning theory, our results showed that servant leadership influences work engagement. Similarly, trust in a leader has a direct impact on work engagement. Furthermore, the moderating role of trust in the leader in a link between servant leadership and work engagement was also explored, and the moderating role was confirmed. Our study shows that trust in leaders boosts work engagement. We hope this study may assist the platform for further studies.

It is vital for leaders, employees, and human resource departments not to ignore the importance of leadership roles and job outcomes. Servant leadership plays an important role in crafting a supportive work environment. Leadership development programs can be designed, which may improve the working conditions and make employees involve in their duties positively. Engaged employees enhance the overall performance. Since the organizational future depends upon employees' positive behavior, the higher authorities need to create an atmosphere of mutual trust and empower employees to work at their best. The Heads of the Universities need to communicate properly about hurdles. In case of crises, the Head is not supposed to detriment the trust, instead needs to communicate and share the problem with the subordinates. Such action may strengthen the trust in the leader, and employees may perform better even in critical spells. Mutual trust unlocks opportunities, and employees understand that mutual trust sustains relationships and keeps individuals viable. By creating a trustworthy environment, employees would be more engaged. Management may reap the benefits by providing a trustworthy environment where the employees feel secure and can work with more enthusiasm.

### Theoretical Contribution

Our study contributes to the literature by following the recommendations suggested by Borst et al. (2020) and Haq et al. (2021). The findings indent leaders boost the feelings of employees, and thus, the result can be more engaged. Remarkably, our findings are consistent with those of the studies conducted in developed economies like North America and Europe. Our findings show that irrespective of the region, employees in Pakistan (an Asian developing country) yielded homologous results. Theoretically, in line with social exchange theory, our results confirmed that employees being supervised by servant leadership were more engaged with a view to recompense for the organization (Blau, 1964). In the current era, monitoring and close supervision are no longer necessary. Instead, facilitating strategies are required to be designed and implemented. It is important since outstanding financial gains are based on engagement.

Engagement cannot be secured without trust. Trust in a leader creates a feeling of safety. In times of organizational stress, the value of feeling comfortable enough to engage is amplified. Trust must be earned, and it might happen fast or not at all, especially for newcomers. It advocates that more care needs to be taken regarding the socialization process of newcomers as they arrive with implied expectations. Individuals learn to trust depending on what occurs to them and what does not happen to them, as well as what occurs to others. It implies that management can secure trust by observing not only what happens to employees but also what occurs around them. A nurturing environment of trust and engagement can be got by acknowledging and expressing sensitivity to employees' needs.

### Limitations and Future Directions

This study is not without limitations. First, this study was single-sourced and conducted at a single organization. Multiple sources and different organizations may be considered for further studies. The causality was not answered. Randall et al. (1999) suggested that longitudinal studies are needed to determine causality. Second, employee work engagement was measured as a single dimension. The literature suggests two types of work engagement, namely, work engagement and organizational engagement (Saks, 2006). Therefore, future studies may consider these two dimensions with other outcomes. Third, trust in a leader was taken as a moderating variable. However, trust has been classified as affective or cognitive trust (McAllister, 1995; Dirks and Ferrin, 2002). Based on the social process, the former refers to an emotional link between leader and subordinate, while the latter is derived from the leader's characteristics (Shuck and Herd, 2011). Thus, both can be distinctively studied. Finally, other individual traits, e.g., personality traits and perception of organizational justice, may be tested with negative behavior (e.g., deviant behavior and knowledge hiding) for moderating and the underlying mechanism.

## Data Availability Statement

The raw data supporting the conclusions of this article will be made available by the authors, without undue reservation.

## Ethics Statement

The studies involving human participants were reviewed and approved by Departmental Ethical Committee, Faculty of Education, University of Malakand, Chakdara, Pakistan. The patients/participants provided their written informed consent to participate in this study.

## Author Contributions

All authors listed have made a substantial, direct, and intellectual contribution to the work and approved it for publication.

## Conflict of Interest

The authors declare that the research was conducted in the absence of any commercial or financial relationships that could be construed as a potential conflict of interest.

## Publisher's Note

All claims expressed in this article are solely those of the authors and do not necessarily represent those of their affiliated organizations, or those of the publisher, the editors and the reviewers. Any product that may be evaluated in this article, or claim that may be made by its manufacturer, is not guaranteed or endorsed by the publisher.
